# Genomic divergence across the tree of life

**DOI:** 10.1073/pnas.2319389122

**Published:** 2025-02-27

**Authors:** Rowan Hart, Nancy A. Moran, Howard Ochman

**Affiliations:** ^a^Department of Molecular Biosciences, University of Texas at Austin, Austin, TX 78712; ^b^Department of Ecology and Evolution, University of Chicago, Chicago, IL 60637; ^c^Department of Integrative Biology, University of Texas at Austin, Austin, TX 78712

**Keywords:** biodiversity, species boundaries, genomic divergence, genetic recombination

## Abstract

Nucleotide sequence data are being harnessed to identify species, even in cases in which organisms themselves are neither in hand nor witnessed. But how genome-wide sequence divergence maps to species status is far from clear. While gene sequence divergence is commonly used to delineate bacterial species, its correspondence to established species boundaries has yet to be explored across eukaryotic taxa. Because the processes underlying gene flow differ fundamentally between prokaryotes and eukaryotes, these domains are likely to differ in the relationship between reproductive isolation and genome-wide sequence divergence. In prokaryotes, homologous recombination, the basis of gene flow, depends directly on the degree of genomic sequence divergence, whereas in sexually reproducing eukaryotes, reproductive incompatibility can stem from changes in very few genes. Guided by measures of genome-wide sequence divergence in bacteria, we gauge how genomic criteria correspond to species boundaries in eukaryotes. In recognized species of eukaryotes, levels of gene sequence divergence within species are typically very small, averaging <1% across protein-coding regions in most animals, plants, and fungi. There are even instances in which divergence between sister species is the same or less than that among conspecifics. In contrast, bacterial species, defined as populations exchanging homologous genes, show levels of divergence both within and between species that are considerably higher. Although no single threshold delineates species, eukaryotic populations with >1% genome-wide sequence divergence are likely separate species, whereas prokaryotic populations with 1% divergence are still able to recombine and thus can be considered the same species.

Species are increasingly being detected based on DNA sequence criteria, with applications extending to assessments of biodiversity, wildlife conservation, and habitat preservation and restoration. Despite a large number of studies that exploit sequence divergence of particular genomic regions as a basis for recognizing species (e.g., 1, 2), there are as yet no broadly applicable conventions regarding benchmarks for differentiating species on the basis of genome-wide divergence. Potentially, these vary greatly due to the particular features and criteria on which species assignments are based or due to biological differences among major groups of organisms. Species have conventionally been recognized based on morphological, behavioral, ecological, or geographic characteristics that are discrete and exclusive to a group of organisms. The expectation is that these identifying features generally conform to genetic discontinuities, although many exceptions are known (3, 4). Molecular methods, such as 16S rRNA gene sequencing in bacteria (5–7) and DNA barcoding or restriction site-associated DNA sequencing (RADseq) in eukaryotes (8–10) have been widely applied for species assignments. Many of these approaches subsample genomic regions through the use of conserved PCR primers or restriction site sequences. More recently, with the introduction of low-cost, short-read sequencing without targeted amplification, environmental sampling of random genomic DNA has become routine (11, 12). For eukaryotes, an increasing number of studies (transcriptome or RNAseq analyses) generate sequences of a large sample of protein-coding regions by selecting transcripts on the basis of their polyadenine tail (e.g., 13, 14). These developments open the possibility of using genome-wide sequence-similarity metrics to identify, distinguish, and discover species.

## Reproductive Isolation and Gene Flow in Eukaryotes and Prokaryotes.

Although many concepts and definitions of species have been proposed, a central element in most is reproductive isolation: Separate species accumulate genetic differences over time whereas gene flow among conspecifics homogenizes the variation within a species. Potentially, species can be defined genetically and in the same manner for all sexual lifeforms (15, 16). However, methods based on only a single gene are less useful when samples consist of highly fragmented DNA, as for environmental DNA samples. These scraps of genomic sequences are frequently insufficient to assess the extent to which sequence divergence enables the recognition of species across the tree of life (11, 17, 18).

In contrast to eukaryotes, prokaryotic diversity is dominated by lineages that show few easily observable distinctions based on morphology, or behavior, or geography. Cultivation requirements and metabolic profiling were long used as a basis for species assignment, but it is now recognized that most prokaryotes resist cultivation (19, 20). As a consequence, genetic characters have been used routinely since the 1970s both for the recognition and classification of bacterial lineages and in the delineation of bacterial species (21, 22). Under current practices, species status is commonly assigned based on nucleotide sequence identity thresholds, often at a genomic level. An average nucleotide identity (ANI) of ≥95% for the set of orthologous genes shared between strains is regularly applied to circumscribe bacterial species (23–26). This threshold often resolves clusters of strains that align with established species-level classifications made prior to the availability of genomic sequences (27).

On account of their asexual mode of reproduction, bacteria were once considered to be strictly clonal and thus exempt from classification based on reproductive isolation and gene exchange (28, 29). However, we now know that homologous recombination is a regular feature of most bacteria: Upwards of 90% of bacterial species undergo some degree of recombination in their core set of genes (15, 30). Thus, most bacteria are amenable to classification into species on the basis of gene flow—i.e., the same criterion regularly used to define eukaryotic species.

In bacteria, homologous recombination can occur through conjugation or through the uptake of DNA released from nearby lysed cells. In either case, the extent of sequence identity between the foreign DNA and the recipient genome is key (31–33). This is because sequence divergence itself directly disrupts homologous exchange, and the requirement for high sequence identity potentially imposes an ANI cutoff. Explicit ANI thresholds have been advocated to circumscribe and differentiate bacterial taxa; however, no single value for sequence divergence delineates bacterial species generally. Under the recombination/gene-flow criterion, some 30% of reasonably well-sampled bacterial species contain members that do not meet the prescribed ANI threshold of ≥95% (15, 30). This variation in intraspecific ANI values is consistent with evidence that homology requirements for recombination vary among bacterial groups (34–36). In sum, almost all bacteria can be assigned to species based on reproductive isolation that is based on sequence divergence, but they cannot be demarcated by a universal ANI value (37).

Eukaryotes also must meet sequence-identity requirements in order for recombination to occur (38–41). However, gene flow in eukaryotes depends on additional features, such as mating type loci in yeast, mating behaviors, compatibility of sperm with female reproductive tracts or pollen with stigma, and compatibility of gametes. Changes in a single locus can initiate reproductive isolation (42, 43) and functional incompatibilities involving as few as two loci can preclude successful sexual crosses (44, 45), despite high identity across other parts of the genome. Consequently, expectations for the minimal ANI that allows gene exchange between members of a bacterial species are unlikely to align with values observed for eukaryotic species.

Genome-wide ANI values and their relation to species status have been previously calculated for prokaryotes using genomic databases (15, 23, 24, 26, 30). Here, we extend this to eukaryotes, in which the requirements for recombination and gene flow can be much more complex. We amass within-species ANI values of orthologous protein-coding genes for eukaryotes representing 25 phyla and consider the minimal ANI values (eukANI*min*) between conspecifics to represent the upper limit of sequence divergence within a species. Using the values established for prokaryotes, we then consider why they differ from those for eukaryotes. Finally, we speculate on the linkage between this metric and assessments of biodiversity.

## The Magnitude of Sequence Divergence within Species.

To estimate sequence identities between conspecifics, we obtained 2717 genome sequences and their corresponding reference sequence data from 762 nominal eukaryotic species. For each species, available genomic sequences were aligned to a taxon-specific set of single-copy orthologs (SCOs) from the reference genome (see Methods), and the minimal ANI value between one of the genomic sequences and the reference genome was extracted (eukANI*min*), i.e., the estimated upper limit of divergergence between conspecifics. This approach was evaluated on simulated genomes from a broad range of eukaryotic species, revealing accuracy within 0.1% for all major taxa except mammals (up to 0.3%). Mammalian sequences were more prone to alignment errors (up to 0.3% and typically overestimating within-species diversity), possibly due to shorter exon lengths and the misannotation of intron boundaries (46), and these artifacts likely lead to overestimation of within-species diversity.

**Fig. 1.**
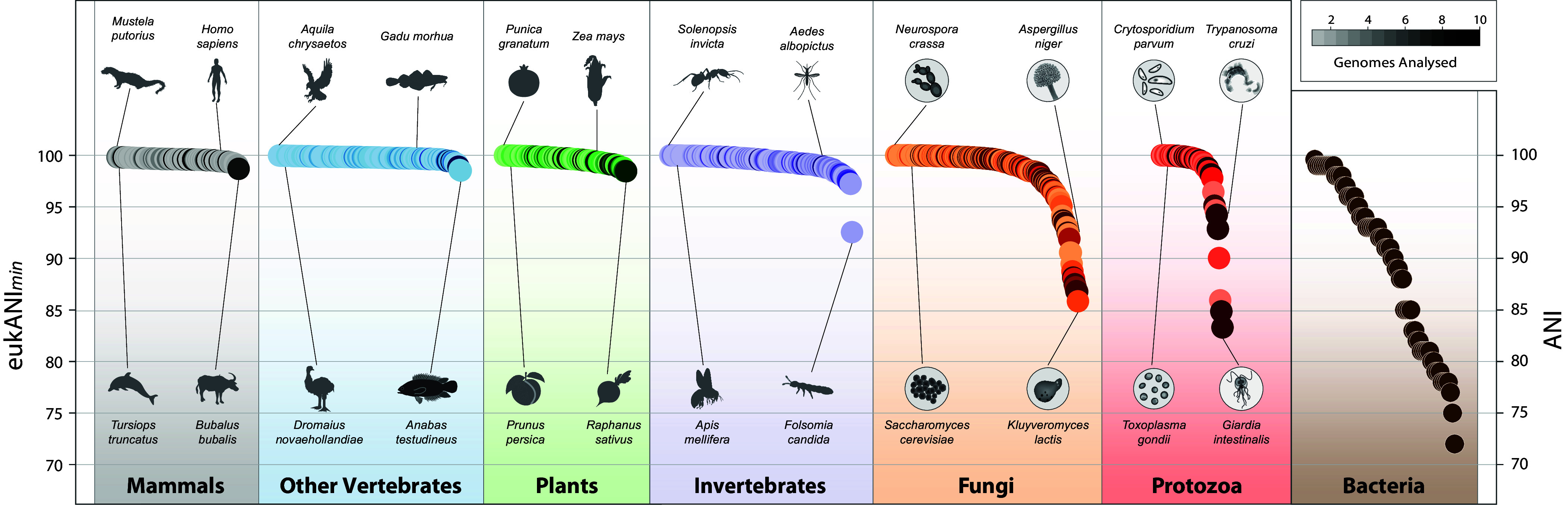
Maximum genomic divergence within species. Each nominal species of eukaryotes is represented by a spot, whose color intensity indicates the sampling depth for that species. Values for each eukaryotic species are expressed as eukANI*min*, calculated as the average nucleotide sequence similarity of orthologous coding regions in the two maximally diverged conspecifics whose genomes are available for a species. For bacteria, ANI values are calculated as the average nucleotide sequence identity for orthologs shared by the two maximally divergent strains within each BSC-defined species, each containing at least 15 sequenced genomes.

Conspecifics within most eukaryotes have high sequence identity, with 633 of the 762 nominal species exhibiting a eukANI*min* ≥ 99% (Fig. 1). Vascular plants and vertebrates have particularly high within-species eukANI*min* values, averaging 99.6% (*n* = 86) and 99.7% (*n* = 254), respectively, and even the outlier species (i.e., those species that encompass the most highly divergent conspecifics) within these groups display a high average eukANI*min* of 98.5%. The narrow range of high eukANI*min* values provides a first indication that the genomic sequence divergence within eukaryotic species is, on average, much lower than in bacteria.

There are few associations of within-species divergence with taxonomic group or life-history features. For example, despite wide variation in ecology, demography, behavior, and social structure among mammals, species within each of the well-sampled orders display a similar range of eukANI*min* values (rodents 99.2 to 99.8%, *n* = 17; primates 99.4 to 99.7%, *n* = 16; bats 99.2 to 99.6%, *n* = 5; carnivores 99.5 to 99.8%, *n* = 21; artiodactyls, 98.7 to 99.7%, *n* = 19). Furthermore, each of the great ape species (*Pan paniscus, Pan troglodytes, Pongo abelii, Gorilla gorilla,* and *Homo sapiens*) has the same eukANI*min* of 99.6%. Similarly, within plants, no well-sampled family [e.g., Fabaceae (legumes); Poaceae (grasses)] stood out as having a significantly higher or lower eukANI*min* value, although the divergence among the better-represented brassicas (*Arabidopsis* and *Brassica* spp.), though still very low, averaged about twice that of other plant families, with mean eukANI*min* values of 99.2 and 99.6 respectively.

Invertebrate species also have high eukANI*min* values, averaging 99.4% (95% CI = ±0.14%, *n* = 135), but, in contrast to vertebrates, they are less uniform, ranging from 97.2 to >99.9%, with a single outlier (*Folsomia candida*) at 92.6%. On average, eukANI*min* is significantly higher in vertebrates (99.7%, CI = ±0.03%, *n* = 254) than in invertebrates (*P* = 1.4e−5, two-tailed *t* test), whose sampling is dominated by insects (*n* = 98). In contrast to metazoans and plants, named species in both fungi and protozoa present a broad range of eukANI*min* values, with eukANI*min* values in fungi ranging from 85.8 to >99.9% (averaging 98.4%, CI = ± 0.36%, *n* = 233) and in protozoans ranging from 83.3 to >99.9% (averaging 98.1%, CI = ± 1.03%, *n* = 54). Notably, 21 currently recognized species of fungi and 7 species of protozoa exhibit eukANI*min* values lower than 95% (Dataset S1).

## Effects of Sampling and Species on Estimates of Conspecific Divergence.

Because sampling effects have been shown to affect other DNA-based approaches (47), we investigated the extent to which variation in eukANI*min* is due to the availability of genomes for each species. The numbers of genomes used in the calculation of eukANI*min* (not including each species’ reference genome) varied from 1 to 10 (averaging 3.3) across the 762 species examined, and overall, there is positive correlation between the number of genomes analyzed (*GA*) and maximal divergence (*P* = 2.0e−12), as assessed by linear regression. However, this correlation explains only a small amount of the variation present in the data (*r*^2^ = 6.3e−2), and after partitioning species into broad taxonomic categories, this correlation loses significance in mammals and protists while maintaining signals in nonmammalian vertebrates, plants, and fungi (*SI Appendix*, Fig. S1).

It is also possible, indeed likely, that some divergent genomes that are designated as the same species in the databases actually represent separate species. We note that this circumstance would eliminate the more divergent members of a designated species and some of the outlying eukANI*min* values, and thereby increase eukANI*min*. The presence of cryptic species could potentially impact ANI values of both bacteria and eukaryotes. However, it is less of an issue in the bacteria used in our analyses, which were previously tested and differentiated based on gene exchange, such that nonrecombining strains (i.e., cryptic species) have been identified (15, 30). Fungi, which display some of the lowest eukANI*min* values, are particularly likely to contain cryptic species, as they can present almost indistinguishable morphologies despite distinct genetic profiles (48, 49). Even among well-studied fungal species, instances of high conspecific divergence are likely to be caused by the presence of cryptic species, as appears to be the case for *Aspergillus fumigatus*, *Aspergillus nige*r and *Cryptococcus neoformans* (50–52). Finally, we acknowledge that the sampling scheme as well as sample size can impact our estimates of eukANI*min* in that genomes sampled locally are likely to be more closely related than genomes sampled throughout a species’ range.

## Life-History and Population-Level Effects on Conspecific Divergence.

Previous studies examining the genetic variation within eukaryotic species focused primarily on levels of neutral variation between conspecifics (53, 54). To derive a metric that is directly comparable to bacterial ANI values, we considered genomic divergence across all sites in the coding regions of all SCOs that are conserved within broad taxonomic groups (i.e., the BUSCO set of genes) (55–58). For very closely related genomes, such as those within most eukaryotic species, measuring divergence across all sites is nearly the same as confining analyses to neutral diversity since there has been insufficient time for selection to act differentially on these sites (59, 60).

Population size can impact levels of polymorphism within species (61–63), but we are focusing on the maximum divergence between conspecifics, measured as eukANI*min*, which depends on divergence time and should be less influenced by population size. Whereas prior work reported little association between actual levels of nucleotide diversity within a species and census population size (53), we find that there is no significant association between population size and eukANI*min* for the 52 metazoan species that overlapped with the dataset reported in ref. 47 (*P* = 0.9, linear regression).

Compared to plants and vertebrates, invertebrates present high variance in eukANI*min* values, potentially reflective, at least in part, of the presence of cryptic species in many invertebrate groups. The outlier among invertebrates, *F. candida* (eukANI*min* = 92.6%, *GA* = 2.0) is mostly parthenogenetic and has recently been shown to contain cryptic species (64), as has *B. tabaci* (65), another named species with a relatively low eukANI*min* (97.4%).

Excluding Entomobryomorpha, whose sole member is *F. candida*, the five invertebrate orders with the lowest average eukANI*min* all consist of marine organisms—Amphioxiformes (lancelets, Chordata), Balanomorpha (barnacles, Arthropoda), Valvatida (starfish, Echinodermata), Temnopleuroida (sea urchins, Echinodermata), and Ostreida (oysters, Mollusca)—that manifest some of the lowest eukANI*min* values among multicellular invertebrates (97.8%, 98.4%, 98.6%, 98.9%, 99.0%, respectively).

The variance in eukANI*min* values in invertebrates could also reflect some reproductive or ecological traits. Among insect orders, Hymenoptera have, on average, significantly higher eukANI*min* values (99.9%, CI = ± 0.07%, n = 17, -*GA* = 2.3) than either Diptera (99.4%, CI = ± 0.17%, n = 52, -*GA* = 2.6) (*P* = 7.2e−7, two-tailed *t* test) or Lepidoptera (99.2%, CI = ± 0.32%, n = 18, -*GA* = 2.4) (*P* = 5.2e−4, two-tailed *t* test). The lower genetic divergences in hymenopteran species, while significant, are slight and could reflect sampling biases, differences in life cycles or mutation rates, or the haploidy and lack of recombination in hymenopteran males.

Within insects, there is no obvious distributional or lifestyle feature common to the five species with the lowest eukANI*min* (*Bemisia tabaci, Drosophila takahashii, Helicoverpa armigera, Plutella xylostella,* and *Teleopsis dalmanni*; whose eukANI*min* are all between 97 and 98%). In some cases, elevated divergence is affected by the presence of cryptic species, as known for *B. tabaci* (65) or by introgression with related species, as known for *H. armigera* (66).

As discussed above, the lower eukANI*min* values observed in fungi might represent species that contain either divergent asexual lineages or cryptic sister species. Nevertheless, the extensive species sampling in these taxonomic groups allows appraisal of eukANI*min* values in relation to reproductive mode, which is considered to influence the amount of variation in a species. Other factors being equal, it takes longer for genes in asexual species to trace to a common ancestor than for outcrossing species of the same size (67), which would yield increased levels of the maximal divergence in asexual species. However, analysis of the effect of reproductive mode on intraspecies divergence is complicated by the potential for many protists and fungal species to reproduce both sexually and clonally. Indeed, some fungi regarded as strictly clonal possess MAT loci, considered an indicator of sexual mating ability (68, 69). Similarly, many protists mix reproductive modes, with some species long thought to reproduce only asexually (*e.g., Leishmania infantum*, *Giardia intestinalis, Trichomonas vaginalis*) showing evidence of sexual recombination or possessing genes involved in meiosis (70, 71).

In fungi, the average eukANI*min* of Ascomycota (98.5%, CI = ±0.41%, *n* = 189, -*GA* = 4.4) is significantly higher (*P* = 4.4e−2, one-tailed *t* test) than that of Basidiomycota (97.6%, CI = ±0.92%, *n* = 35, -*GA* = 4.5), which is perhaps indicative of systemic differences in outcrossing frequency, though this phylum-level difference could be attributable to additional factors. Since few, if any, fungi are exclusively clonal (68, 72, 73), we questioned the effects of outcrossing on the divergence among conspecifics by examining the relationship between eukANI*min* and the degree of linkage disequilibrium (LD) in fungal species whose genetic population structures have been evaluated (74). Based on these 10 species, there is no significant association between genomic divergence and LD (*r* = 0.36, *P* = 0.31; Spearman’s rho), though admittedly, LD is an imperfect measure of the degree of outcrossing. Extending this analysis to include 12 fungal species for which estimates of outcrossing frequency are available (72) yielded no association between intraspecies divergence and reproductive mode.

## The Extent of Sequence Divergence Between Sister Species.

**Fig. 2.**
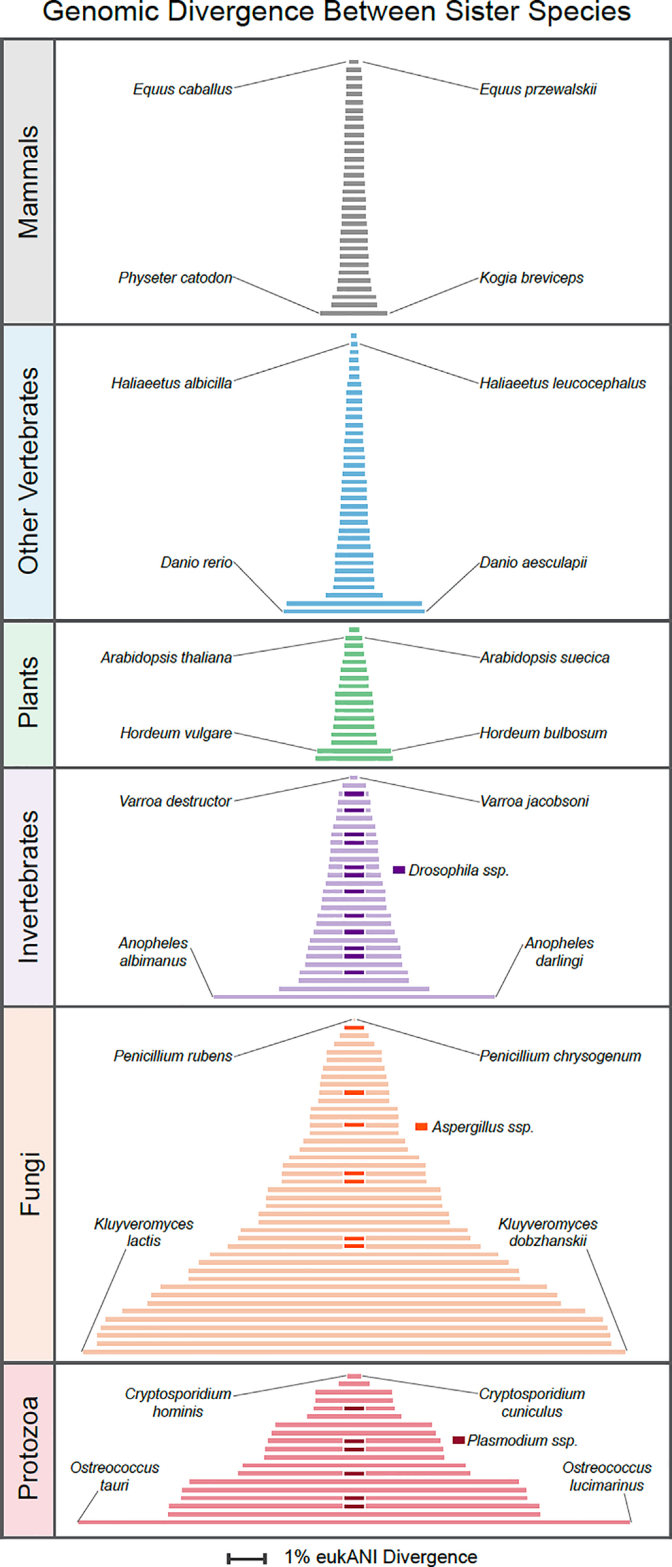
Genomic sequence divergence between reference genomes of sister taxa in major groupings of eukaryotes. Within each group, the pairs of sister taxa presenting extreme values are labeled, and within invertebrates, fungi, and protozoa, bars with darkened central segments denote different sister-species pairs from the same genus.

Genome-wide nucleotide sequence similarity thresholds, despite shortcomings with their application, are regularly used to delineate species boundaries in bacteria. However, the utility of such thresholds for distinguishing eukaryotic species has not been fully established, despite their potential impact on sequence-based biodiversity studies. Measuring the sequence divergence (eukANI) between 173 pairs of sister species representing 65 orders of eukaryotes, we find that the degree of sequence similarity between species varies considerably across taxonomic groups and is not consistent for species within a genus (Fig. 2). This variation is expected: The speciation events giving rise to modern sister-species pairs occurred at different times in the past.

In animals, sister species within genera of homeotherms (birds and mammals) average 99.4% eukANI (range 98.2 to 99.9%, *n* = 44), ectothermic vertebrate sister species average 99.0% eukANI (range 96.2 to 99.8%, *n* = 23), invertebrate sister species average 98.1% eukANI (range 92.3 to 99.9%, *n* = 28), and protozoan sister species average 94.2% eukANI (range 84.8 to 99.7%, *n* = 19). The average eukANI of sister species of plants, like those of vertebrates, are high, averaging 99.1% (range 97.9 to 99.8%, *n* =17), whereas fungi mirror protists, averaging 94.3% eukANI (range 85.0 to >99.9%, *n* = 42).

To resolve which species are sister taxa, we searched the literature for robust species-level phylogenies for each group (Dataset S2). In general, the phylogenies available for vertebrates were more comprehensive than those for invertebrates (75, 76). Because the identification of sister species depends on taxonomic sampling, it is possible that we are missing the true sister species for some groups, which would lead to an overestimation of the divergence between the presumed sister species. The eukANI values will be dictated by the age of the speciation event, such that a value measured for a contemporary pair will generally be lower than the eukANI when they first achieved species status. Estimated values could also be impacted by sampling, the presence of cryptic species, or rates of sequence evolution.

## Species Cohesion and Discontinuity.

One of most salient features concerning species formation emerges from comparisons of the maximum sequence divergence within a species (eukANI*min*) to the sequence similarity (eukANI) to its sister species. In humans, for example, the within-species eukANI*min* is 99.6%, and the sequence similarity to our closest extant relative, *P. troglodytes*, based on the identical gene set is 99.1%, yielding a genetic discontinuity of 0.5% between these sister species.

The genetic relationships and hybridization potential among members of the most well-sampled genus in our dataset, *Drosophila*, are fairly well established. Among the 12 sister-species pairs examined, *Drosophila persimilis* and *D. pseudoobscura*, which hybridize at low levels in areas of sympatry (77, 78) display the lowest degree of discontinuity (eukANI*min* of 99.6% and between-species eukANI of 99.2%, giving discontinuity of 0.4%). These values are similar to those estimated for the human–chimpanzee comparison.

Except for very recently diverged species, conspecifics are expected to be more closely related to one another than to individuals classified as different species, and distance-based methods of DNA taxonomy typically assume a gap between within-species sequence identity and between-species identity (2). For many cases, however, the genetic distinctiveness between members of the same species and members of different species is not clear-cut. We find low absolute genetic discontinuity (≤0.1%) in 23 sister-species pairs, with some species displaying greater maximal divergence with a member of the same species than with its sister species. In many cases of low discontinuity (17 of 23), there is high sequence similarity between sister taxa (eukANI > 99%), potentially resulting from hybridization, recent divergence, or subspecies that have been awarded status as separate species. The remaining species pairs with low genetic discontiuity are fungi, each exhibiting large divergence between conspecifics (eukANI*min* ≤ 99%). In addition, there are four fungal and two protozoan species with strong negative discontinuity (≤–0.1%), a pattern that suggests the presence of cryptic species. Among mammals, there is strong negative discontinuity (–0.3%) between the northern and common bushtail oppossums, *Trichosurus arnhemensis,* and *T. vulpecula*; however, this is possibly an artifact stemming from the incompleteness of the *T. arnhemensis* genome.

Negative discontinuity is also expected in cases of paraphyletic species, in which divergence of homologous sequences within a species may have preceded divergence with sequences of another species. Many such cases of paraphyly have been demonstrated (e.g., 79), and its overall occurrence has been estimated at about 10% of animal species (80), with a similarly high frequency in plants (81). Following species origin, paraphyly will disappear over time, with the rate depending on generation time and population size. Eventually, barring further species origins, two monophyletic species will emerge, with eukANI*min* for each potentially greater than their pairwise eukANI (82).

## Building Barriers Among Biological Species.

Assigning species boundaries to all lifeforms [possibly even viruses; (83)] based on a uniform and objective criterion would simplify classification and also would allow direct comparisons of the genetic and phenotypic composition of species. Despite the operational appeal of applying DNA-similarity thresholds to assign organisms to the same species, the span of genetic divergence within and between biological species precludes such a practice for any broad taxonomic group. Among eukaryotes, we find few well-sampled orders (or even genera) for which a single ANI value consistently defines species boundaries. This is further complicated by numerous examples of species for which the reference genome is more divergent from genomes of conspecifics than from that of a sister species. Determining whether these species are examples of cryptic speciation, paraphyly, or other factors may change our understanding of the utility of ANI in defining species boundaries.

The universality of defining species based on gene exchange and reproductive incompatibility offers numerous clues about the manner in which the genetic changes that disrupt gene flow arise and species are formed. In sexually reproducing organisms, the genetic mechanism by which reproductive isolation can be achieved is most appealingly postulated in the Dobzhansky–Muller model, which requires the occurrence of only two loci that possess alleles that are incompatible when combined (45, 84). Reproductive incompatibilities can also be attained through interactions among multiple loci when each confers a partial effect (85, 86). This model might explain why, for most eukaryotic species, both conspecifics and sister species are very closely related, with most having ANI values much higher than 99%.

Bacteria contrast with eukaryotes, in that the accumulation of sequence changes serves as the primary basis for suppressing recombination and homologous exchange—thereby prohibiting gene flow and generating barriers between species (33–35, 87). Because bacteria differ in their homology requirements for recombination, some bacterial species encompass more genetic divergence than others. However, because sequence divergence itself forms the barriers between bacterial species, genetic discontinuities between species are established and maintained.

Bacteria can sample DNA from their surroundings, and, if sufficiently similar, it can be recombined into their genomes. This leads to the situation in which different genomic regions may “speciate” at different times, with highly conserved regions such as the ribosomal RNA operons continuing to recombine after other regions have become isolated (88, 89). An interesting implication is that a long-favored view of speciation in eukaryotes, as arising from geographic barriers (90), may apply with few or no exceptions to bacteria, if geographic barriers are taken to include small-scale spatial separation by niche. Bacterial species coherence requires exposure to DNA released by other members of the species, so a shift to a new spatial niche, such as a new host or new site of colonization, can trigger species formation (91).

EukANI values, though not reliable indicators of species status or membership, can serve as guides in the broad differentiation of species. For example, eukANI values <99% generally indicate distinct species of vascular plants and vertebrates, as we found that <3% of 340 species included conspecifics that diverged beyond this value. This threshold is conservative and would not flag all cases of novel species, as we observed many cases in which genomes with eukANI > 99% represent distinct species. Vertebrates and plants are currently the focus of many conservation efforts, and application of such sequence-based thresholds could serve as a conservative guideline for recognizing the likely presence of a distinct species.

## Materials and Methods

### Genomes Analyzed, Taxonomic Classifications, and Core Gene Sets.

We obtained a total of 2717 eukaryotic genome sequences from GenBank (ncbi.nlm.nih.gov/genbank/) and 774 coding sequence (CDS) datasets from RefSeq (ncbi.nlm.nih.gov/refseq/) representing a total 905 nominal species. Genome taxonomies were amassed using the “lineage” function of TaxonKit (92), which organizes the taxonomic information associated with each genome in the NCBI database (ncbi.nlm.nih.gov/taxonomy). Additionally, we downloaded the 67 eukaryotic “odb10” datasets from the Benchmarking Universal Single-Copy Orthologs (BUSCO) website (busco.ezlab.org), which specifies the SCOs common to members of selected taxonomic group (56, 57).

### Eukaryotic Average Nucleotide Identity (eukANI) metric.

As a measure of the genome divergence between organisms, we calculated ANI of coding regions for SCOs in the BUSCO gene set for a given group of genomes. Pairwise comparisons were performed between a coding sequence (‘the reference”) and a genomic sequence (‘the query”) via a three-step process that entailed exome estimation of the query, assembling the set of SCOs from the reference, and calculation of eukANI values as follows:

(i) *Exome estimation*. Each reference coding sequence was searched against the query genome using the blastn function in BLAST+ (version 2.13.0) (https://ncbiinsightsncbi.nlm.nih.gov/2022/03/29/blast-2-13-0/) applying the following parameters: max_target_seqs = 1, perc_identity = 80, evalue = 1e−50, ungapped = true, with the remaining parameters assigned to default settings. Genomic regions with a match to the reference were extracted from the query using samtools (v1.15.1) and culled of redundancies and any genomic fragments smaller than 100 bp in length. Remaining exon fragments, constituting the exome of an organism, were compiled into a single file and compared to the reference coding sequence. Exomes for which the total length was ≤10% of that of the reference were removed from analyses, with the exception of *Trichosurus arnhemensis* and *Pongo pygmaeus* for which available genome sequences are only partially represented.
(ii) *Single-copy ortholog identification*. To assemble the set of SCOs shared between the reference and the query, we made pairwise comparisons, selecting the lowest BUSCO taxon odb10 dataset that contained both organisms. The corresponding orthologs were then identified in the reference using tblastn and extracted with samtools.
(iii) *eukANI calculation*. Exome of the query was searched against the SCOs in the corresponding reference with blastn using the following parameters: max_target_seqs = 1, evalue = 1e−50, ungapped = true, with the remaining parameters assigned to default settings. Mean nucleotide identity values across all exon fragments that mapped ≥95% of their length to a SCO in the reference were calculated for all pairs of genomes. After generating ANI values for a given species, we selected the minimal ANI value (eukANI*min*) to represent the magnitude of sequence divergence within a species.

The reliability of these methods was tested on simulated genomes developed with simuG (93) by assessing the accuracy of eukANI compared to true ANI values across a broad range of eukaryotic species. Alignment errors caused minor deviations from the true values, but for most taxonomic groups, the observed errors were >0.1% for values ranging from 95 to 100% ANI. Mammals, however, tended to have slightly more alignment errors than other taxonomic groups (up to 0.3%), causing overestimation of diversity in simulations with >99% ANI.

### Within-Species Comparisons.

We computed within-species eukANI for all species represented by at least two genomes in GenBank and an available coding sequence in RefSeq, as of February 2022. For species with 10 or fewer genomes in GenBank, we calculated the eukANI between the RefSeq reference and all other genomes, and for species with more than 10 genomes, we randomly subsampled 10 genomes. To characterize the scale of sequence divergence harbored by a species, we selected the lowest pairwise eukANI (maximally divergent, eukANI*min*) value between conspecifics to represent within-species divergence. Except where noted, when contrasting eukANI*min* values between taxa or organismal features, we limited comparisons to groups containing at least nine species or species-pairs to avoid sampling biases.

### Sister-Taxa Identification and Between-Species eukANI Comparisons.

To estimate the sequence divergence between the genomes of sister species, we searched the RefSeq database for genus- or family-level taxa. An organism was considered for analysis if it either had a distinct sister species with an available genome sequence or was sister to a single outgroup clade of two species. In the latter case, we selected the species from within the outgroup that had the most genomic information available. To obtain between-species eukANI values, we compared the CDS of the reference genome of a species to the reference genome sequence of its sister species using the procedures described above.

## Supplementary Material

Appendix 01 (PDF)

Dataset S01 (XLSX)

Dataset S02 (XLSX)

## Data Availability

All study data are included in the article and/or supporting information.
