# Supplementary material for: Genomic divergence across the tree of life
Source: Proc Natl Acad Sci U S A. 2025 Feb 27;122(10):e2319389122. doi: 10.1073/pnas.2319389122 (PMC11912424; doi:10.1073/pnas.2319389122)
Supplement: Supplementary file 1 — Appendix 01 (PDF) [file pnas.2319389122.sapp.pdf]

PNAS

Supporting Information for

Genomic divergence across the tree of life

Rowan S. Hart, Nancy A. Moran, Howard Ochman

Corresponding author: Howard Ochman
Email: howard.ochman@austin.utexas.edu

This PDF file includes:

Supplemental Figure S1

Other supporting materials for this manuscript include the following:

Supplemental Dataset S01. List of species, taxonomic classifications, and genomes analyzed to assess within species variation.

Supplemental Dataset S02. List of taxa, accession numbers and links to reference phylogenies used to assess genomic divergence between sister taxa.

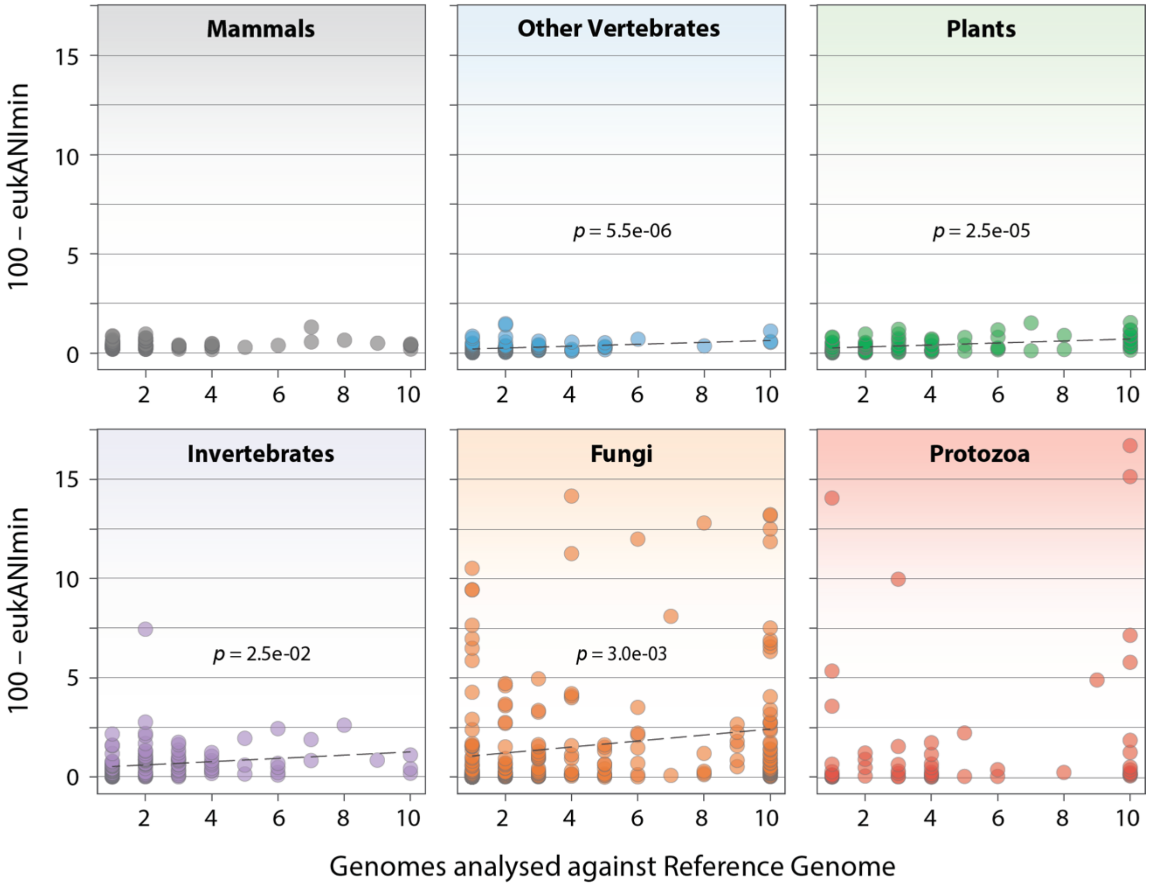

Supplemental Figure S1. Effect of sample size on $eukANI_{min}$. Nominal species are represented by a spot, the y-axis represents divergence (100 – $eukANI_{min}$), and the x-axis shows the number of genomes analyzed. Regression lines are shown for plots with statistical significance (p < 0.05).
